# *Artemisia annua* extract prevents ovariectomy-induced bone loss by blocking receptor activator of nuclear factor kappa-B ligand-induced differentiation of osteoclasts

**DOI:** 10.1038/s41598-017-17427-6

**Published:** 2017-12-11

**Authors:** Sun Kyoung Lee, Hyungkeun Kim, Junhee Park, Hyun-Jeong Kim, Ki Rim Kim, Seung Hwa Son, Kwang-Kyun Park, Won-Yoon Chung

**Affiliations:** 1 Department of Oral Biology, Oral Cancer Research Institute, BK21 PLUS Project, Yonsei University College of Dentistry, Seoul, 03722 Korea; 2 Department of Applied Life Science, The Graduate School, Yonsei University, Seoul, 03722 Korea; 3 Department of Dentistry, Graduate School, Yonsei University, Seoul, 03722 Korea; 4 Department of Dental Hygiene, Kyungpook National University, Sangju, 37224 Korea; 5 Department of Dental Hygiene, Gangdong College, Icheon, 27600 Republic of Korea

## Abstract

The activities of osteoclasts and osteoblasts are balanced to maintain normal bone density. Many pathological conditions cause osteoclastic bone resorption in excess of osteoblastic bone formation, resulting in osteoporosis. We found that oral administration of *Artemisia annua* ethanol extract (AaE) or major components, artemisinin and arteannuin B, to ovariectomized (OVX) mice prevented bone loss, as verified by examining three-dimensional images and bone morphometric parameters derived from microcomputed tomography analysis, as well as serum levels of bone turnover markers and proinflammatory cytokines. The administered doses were not toxic to the liver or kidney and showed promising effects that were comparable to those of 17β-estradiol treatment. At non-cytotoxic concentrations, AaE and active components, artemisinin, artemisinic acid, and arteannuin B, potently inhibited receptor activator of nuclear factor kappa-B ligand (RANKL)-induced osteoclastogenesis and the formation of osteoclast-mediated resorption pits. Furthermore, AaE, artemisinin, and arteannuin B remarkably reduced the expression of the c-Fos and NFATc1 transcription factors, which play critical roles in RANKL-induced osteoclast differentiation. Taken together, the *in vivo* anti-osteoporotic activity of AaE may be derived from the anti-osteoclastic and anti-bone resorptive activities of its active components. AaE has beneficial applications for the prevention and inhibition of osteoporosis and osteoclast-mediated bone diseases.

## Introduction

Bone homeostasis is maintained by osteoclasts and osteoblasts^1^. Osteoclasts, which are derived from monocytes and macrophages, degrade the bone matrix^2^. Mesenchymal stem cell-derived osteoblasts rebuild the resorbed bone and regulate osteoclast differentiation via receptor activator of nuclear factor kappa-B ligand (RANKL) and osteoprotegerin (OPG) expression^3^. When exposed to pathological conditions, such as estrogen deficiency, inflammation, and metastatic cancer, osteoclastic bone resorption surpasses osteoblastic bone formation^4,5^. This excessive osteoclast activity causes many bone diseases, including osteoporosis, rheumatoid arthritis, periodontal disease, and cancer-induced osteolysis^6–8^. Postmenopausal osteoporosis is an important public health problem worldwide^9^.

Most therapeutic agents for the treatment of osteoporosis and other bone diseases are designed to inhibit bone resorption by osteoclasts or stimulate bone formation by osteoblasts^10^. Unfortunately, these therapeutic agents have considerable side effects. The long-term use of bisphosphonates can lead to osteonecrosis of the jaw, nephrotoxicity, and atypical subtrochanteric femoral fractures, and estrogen replacement therapy can elevate the risk of cardiovascular disease and breast cancer^11–13^. Transient increases in serum and urine calcium levels have been reported in patients treated with teriparatide. Furthermore, denosumab, a full human monoclonal antibody against RANKL, is contraindicated in patients with hypocalcemia because it can reduce serum calcium level^14–16^. Therefore, it is necessary to find new agents for the management of osteoporosis.

Medicinal plants with multiple active components have been used to treat a variety of diseases in Asian countries^17–20^. *Artemisia annua* L. (Asteraceae) is a traditional herbal medicine with a long history of safe use for the treatment of malaria. It is the source of artemisinin, an anti-malarial agent with a sesquiterpene trioxane lactone^21^. Because of the presence of flavonoids, essential oils, and sesquiterpene lactones, *A. annua* has anti-oxidant, anti-cancer, anti-microbial, anti-adipogenic, and anti-inflammatory activities^22–26^. Many studies have used artemisinin, isolated from *A. annua*, and its natural and semi-synthetic derivatives to develop more effective anti-malarial agents and to find new treatments for other diseases, including cancer^27–30^.

In the present study, we investigated the protective effects of *A. annua* extract and its active components on bone loss in ovariectomized (OVX) mice. We further examined the inhibitory effects of *A. annua* extract, artemisinin, artemisinic acid, and arteannuin B on osteoclast formation and the expression of related transcription factors, as well as the function of mature osteoclasts, in RANKL-treated mouse bone marrow-derived macrophages (BMMs).

## Results

### A. *annua* extract and its active components inhibited bone loss in OVX mice

**Figure 1:**
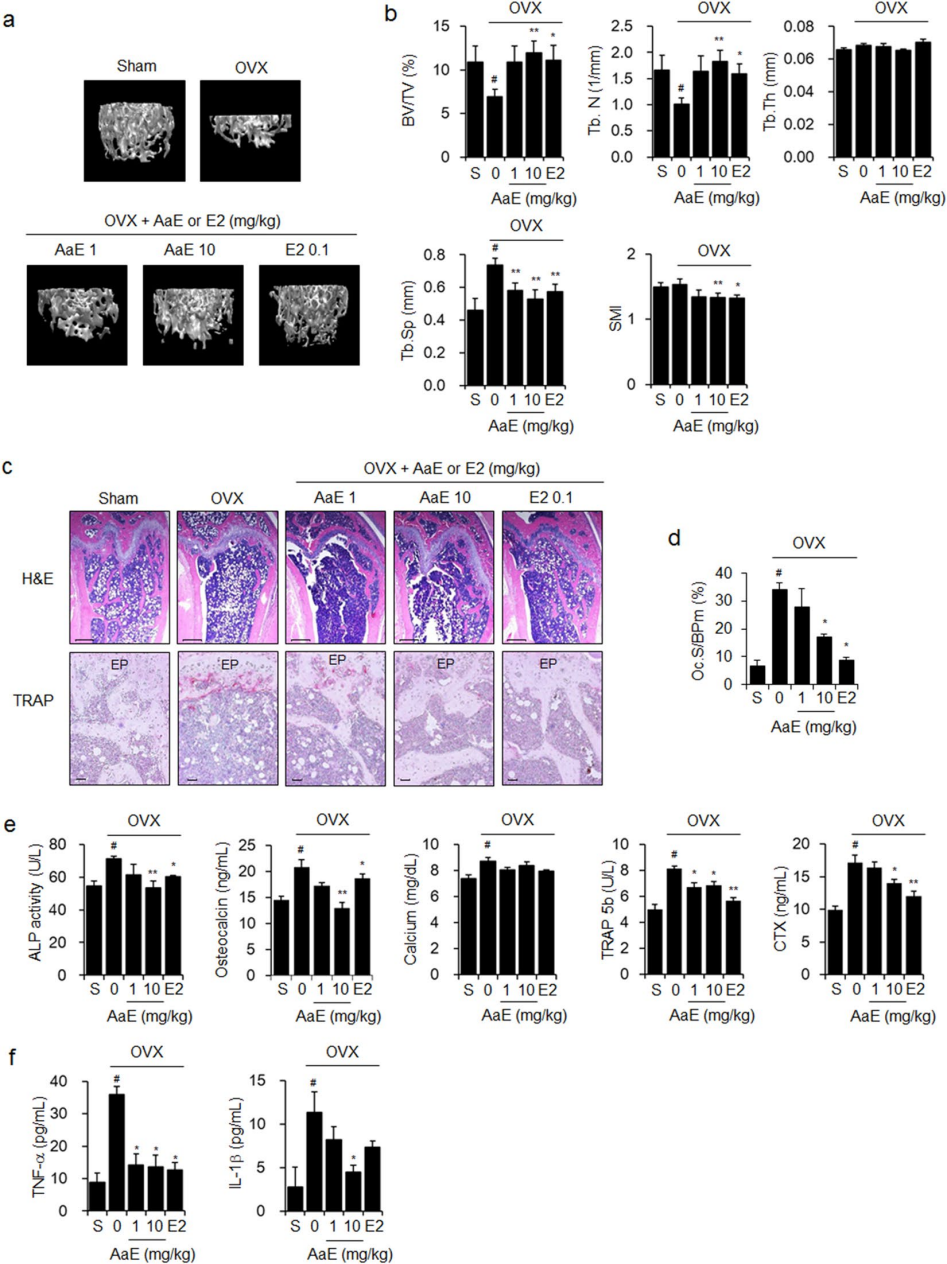
AaE inhibits OVX-induced bone loss in mice. Eight-week-old female OVX mice (N = 10) were administered vehicle (PBS containing 1% DMSO and 1% Tween-20), 1 or 10 mg/kg BW of AaE, or 0.1 mg/kg BW of E2 by oral gavage 5 times per week for 12 weeks. Sham-operated mice (N = 10) received the vehicle alone. (**a**) The trabecular bones of the distal femora of mice were scanned using μCT at 12 weeks, and 3D images were obtained as described in the *Methods* section. One representative image is shown per group. **(b**) Bone morphometric parameters of the mouse femora, including BV/TV, Tb.N, Tb.Th, Tb.Sp, and SMI, were measured using μCT. (**c**) H&E and TRAP staining were performed in the sectioned femoral tissues. EP: epiphyseal plate. Scale bar: 0.396 mm for H&E staining and 10 μm for TRAP staining. (**d**) Osteoclast surface per bone perimeter (Oc.S/BPm) values were calculated from TRAP-stained femoral sections. (**e**) Serum levels of bone turnover markers and (**f**) the pro-inflammatory cytokines TNF-α and IL-1β were quantified using their respective assay kits as described in the *Methods* section. The data are expressed as the mean ± SE. ^#^ *P* < 0.01 *versus* sham-operated mice (S), ^*^ *P* < 0.05, ^**^ *P* < 0.01 *versus* OVX mice.

We first assessed the anti-osteoporotic effects of *A. annua* ethanol extract (AaE) in OVX mice, an animal model of estrogen deficiency-induced osteoporosis. Oral administration of AaE did not suppress OVX-induced weight gain, but the positive control, 17β-estradiol (E2), did (Supplementary Fig. S1a). Three-dimensional (3D) images of femora that were reconstructed using microcomputed tomography (μCT) analysis clearly showed that OVX-induced trabecular bone loss was inhibited by AaE treatment in a dose-dependent manner (Fig. 1a). Bone morphometric parameters, including bone volume over total volume (BV/TV) and trabecular number (Tb.N), were decreased, whereas trabecular separation (Tb.Sp) was increased in the distal femora of the OVX-mice compared with the sham-operated mice. OVX-related changes in bone morphometric parameters were markedly suppressed by AaE treatment. Statistically significant alterations in trabecular thickness (Tb.Th) and the structure model index (SMI) were not detected in the femora of these OVX mice regardless of AaE treatment (Fig. 1b). H&E and TRAP staining indicated that AaE treatment inhibited OVX-induced bone loss and the formation of TRAP-positive osteoclasts on the bone surfaces (Fig. 1c,d). The serum levels of bone turnover markers, including alkaline phosphatase (ALP), osteocalcin, calcium, tartrate-resistant acid phosphatase 5b (TRAP 5b), and C-terminal cross-linking telopeptide of type Ι collagen (CTX), were increased in the OVX-mice. Oral administration of AaE significantly reduced the levels of these osteoporosis-related serum markers (Fig. 1e). Moreover, AaE administration to OVX mice inhibited the increase in the serum levels of tumor necrosis factor (TNF)-α and interleukin (IL)-1β, which are proinflammatory cytokines that play critical roles in estrogen deficiency-induced bone loss^31^ (Fig. 1f).

**Figure 2:**
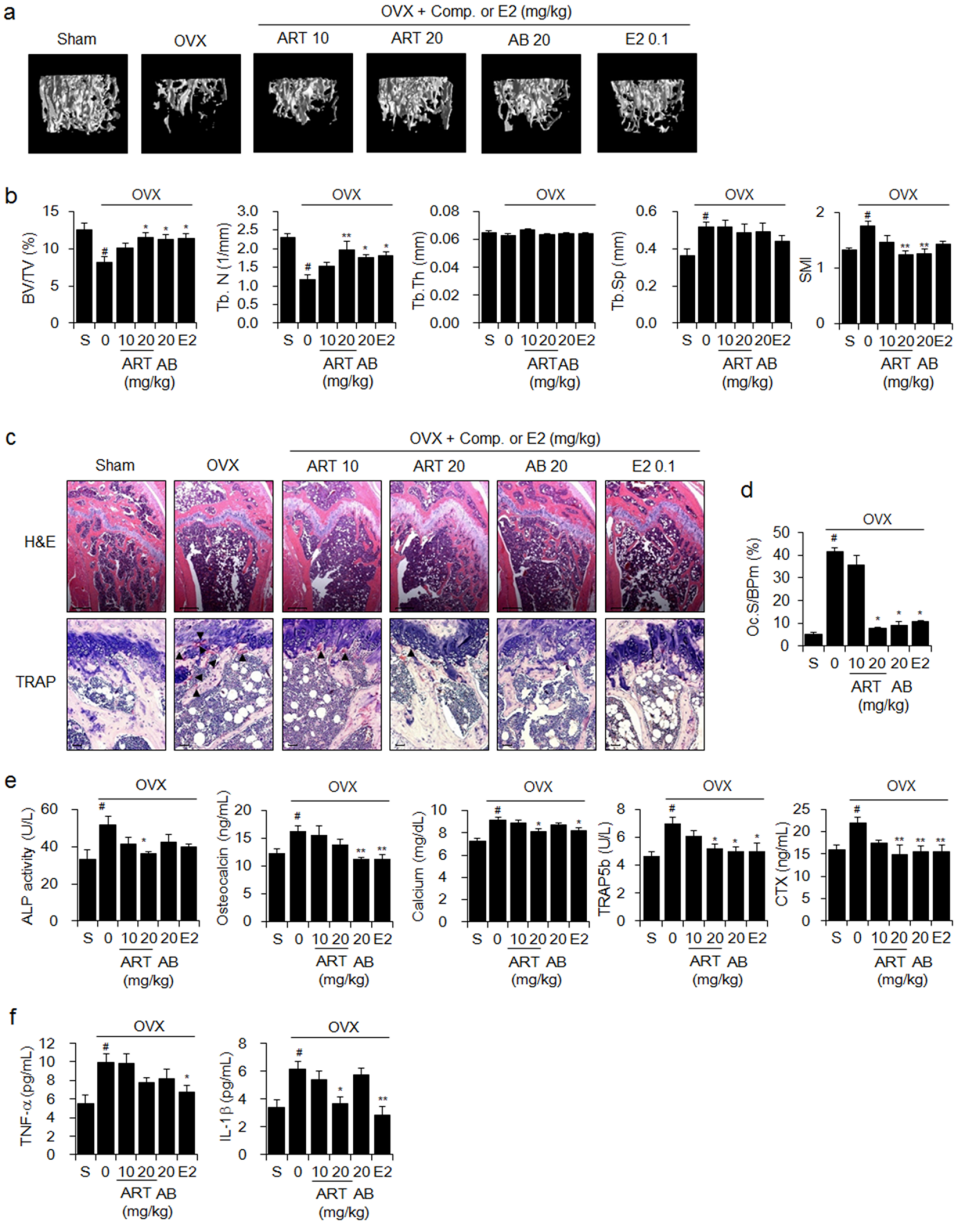
Artemisinin and arteannuin B reduce OVX-induced bone loss in mice. Eight-week-old female OVX mice (N = 10) were administered vehicle (PBS containing 1% DMSO and 1% Tween-20), 10 or 20 mg/kg BW of artemisinin (ART), 20 mg/kg BW of arteannuin B (AB) or 0.1 mg/kg BW of E2 by oral gavage 5 times per week for 12 weeks. Sham-operated mice (N = 10) received the vehicle alone. (**a**) 3D images of the trabecular bones in the distal femora of mice were obtained using μCT at 12 weeks as described in the Methods section. (**b**) Bone morphometric parameters of the mouse femora, including BV/TV, Tb.N, Tb.Th, Tb.Sp, and SMI, were measured using μCT. (**c**) H&E and TRAP staining were performed in the sectioned femoral tissues. Arrowhead: TRAP-positive osteoclast. Scale bar: 0.396 mm for H&E staining and 10 μm for TRAP staining. (**d**) Osteoclast surface per bone perimeter (Oc.S/BPm) values were calculated from TRAP-stained femoral sections. (**e**) Serum levels of bone turnover markers and (**f**) the pro-inflammatory cytokines TNF-α and IL-1β were quantified using their respective assay kits as described in the Methods section. The data are expressed as the mean ± SE. ^#^ *P* < 0.01 *versus* sham-operated mice (S), ^*^ *P* < 0.05, ^**^ *P* < 0.01 *versus* OVX mice.

Next, we verified that orally administered artemisinin or arteannuin B, major components of AaE, significantly inhibited OVX-induced weight gain (Supplementary Fig. S1b) and OVX-induced trabecular bone loss, as shown by 3D images of femora (Fig. 2a), bone morphometric parameters (Fig. 2b), H&E and TRAP staining (Fig. 2c,d), and the serum levels of bone turnover markers (Fig. 2e) and proinflammatory cytokines (Fig. 2f).

As expected, the treatment with AaE, artemisinin, or arteannuin B did not result in elevated serum levels of alanine aminotransferase (ALT) and aspartate aminotransferase (AST), which are indicative of liver damage (Supplementary Fig. S1c), or of blood urea nitrogen (BUN) and creatinine, which are indicative of kidney damage (Supplementary Fig. S1d).

### *A*. *annua* extract suppressed RANKL-induced osteoclast formation and function

**Figure 3:**
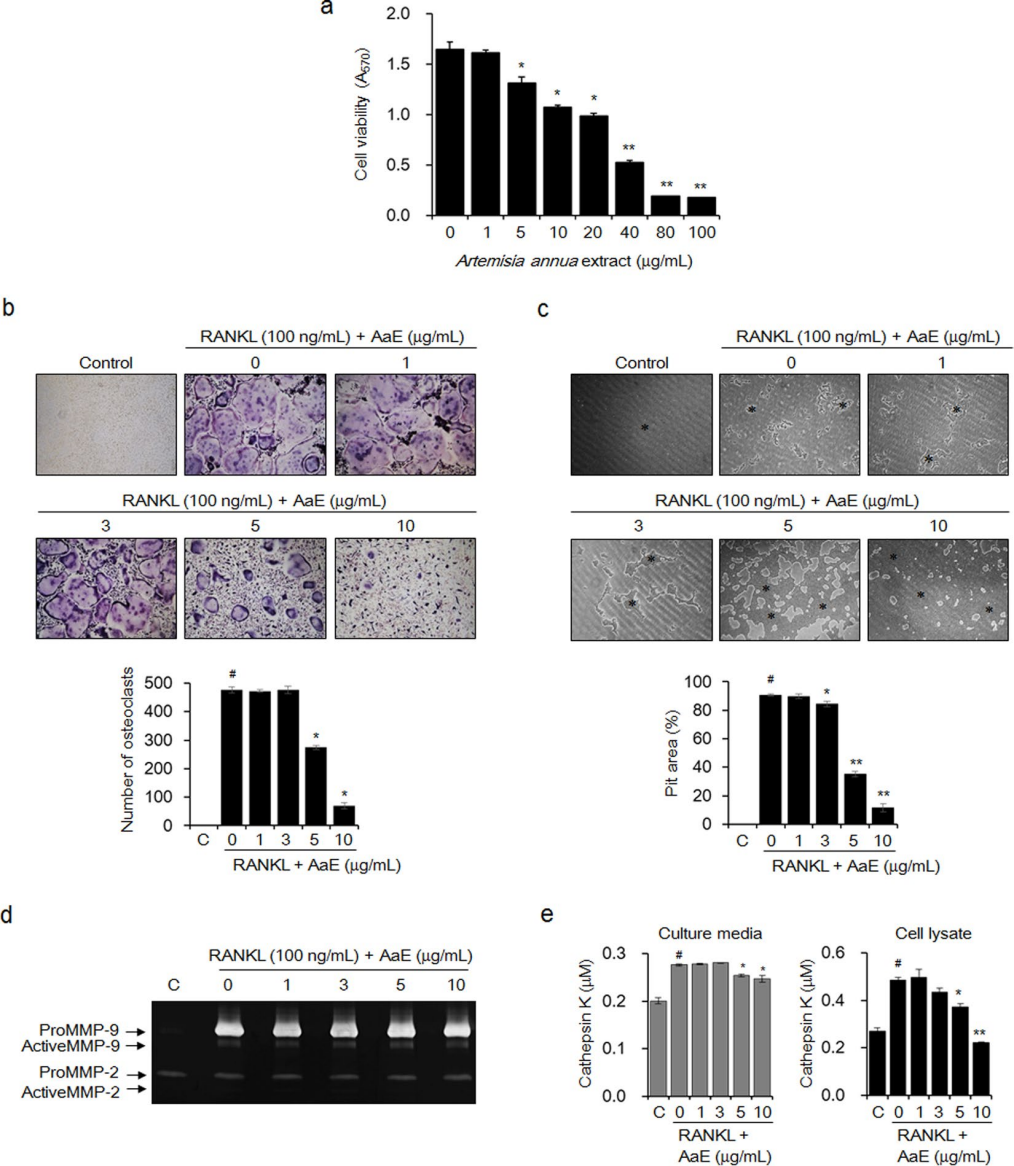
AaE suppresses RANKL-induced osteoclastogenesis and the function of mature osteoclasts. (**a**) BMMs were cultured in α-MEM containing M-CSF (30 ng/mL) and AaE at the indicated concentrations for 5 days. Cell viability was measured with an MTT assay. The data are expressed as the mean ± SE of triplicate experiments. ^*^ *P* < 0.05, ^**^ *P* < 0.01 *versus* BMMs without AaE. (**b**) BMMs were incubated in α-MEM with M-CSF (30 ng/mL), RANKL (100 ng/mL), and the indicated concentrations of AaE for 5 days. The differentiated osteoclasts were detected by TRAP staining, and TRAP-positive multinucleated cells with more than 3 nuclei were counted using light microscopy (magnification, ×100). (**c**) The BMMs were cultured in α-MEM containing M-CSF (30 ng/mL) and RANKL (100 ng/mL) onto Osteo Assay Surface Plates for 5 days. The cells were then treated with the indicated concentrations of AaE for an additional 2 days. The resorption pits (*) were observed using light microscopy (magnification, ×100), and the resorbed area was calculated using ImageJ software. (**d**) MMP-2 and MMP-9 activities in the culture media of osteoclasts were detected by gelatin zymography as described in the *Methods* section. (**e**) Cathepsin K activity in the culture media and lysates of the osteoclasts was measured using a commercially available cathepsin K assay kit according to the manufacturer’s instructions. The images are representative, and the data are expressed as the mean ± SE of triplicate experiments. ^#^ *P* < 0.01 *versus* BMMs without RANKL (C), ^*^ *P* < 0.05, ^**^ *P* < 0.01 *versus* BMMs with RANKL alone.

To examine the effects of AaE on osteoclast differentiation and activity, we identified non-cytotoxic concentrations of AaE using BMMs, which are osteoclast precursors, via an MTT assay. The viability of the AaE-treated BMMs was 79.7% at 5 μg/mL and 65.2% at 10 μg/mL (IC_50_ = 27.1 μg/mL) (Fig. 3a). When the BMMs were treated with RANKL in the presence of AaE for 5 days and then stained for TRAP, osteoclast formation was inhibited by 42.4% at 5 μg/mL and by 85.2% at 10 μg/mL (Fig. 3b). The anti-bone resorptive activity of AaE was assessed by measuring the area of the pits produced by RANKL-induced mature osteoclasts in plates coated with bone mimetic material. AaE markedly reduced the area of the resorption pits by 64.8% at 5 μg/mL and by 88.3% at 10 μg/mL (Fig. 3c). Gelatin zymography indicated that AaE did not inhibit the activities of matrix metalloproteinase (MMP)-2 and MMP-9 in the culture media of the RANKL-induced mature osteoclasts (Fig. 3d). Cathepsin K activity was decreased by 11.2% and 54.3% in the culture medium and lysate, respectively, of the RANKL-induced mature osteoclasts treated with 10 μg/mL AaE (Fig. 3e).

### Artemisinin, artemisinic acid, and arteannuin B interrupted RANKL-induced osteoclast formation

**Figure 4:**
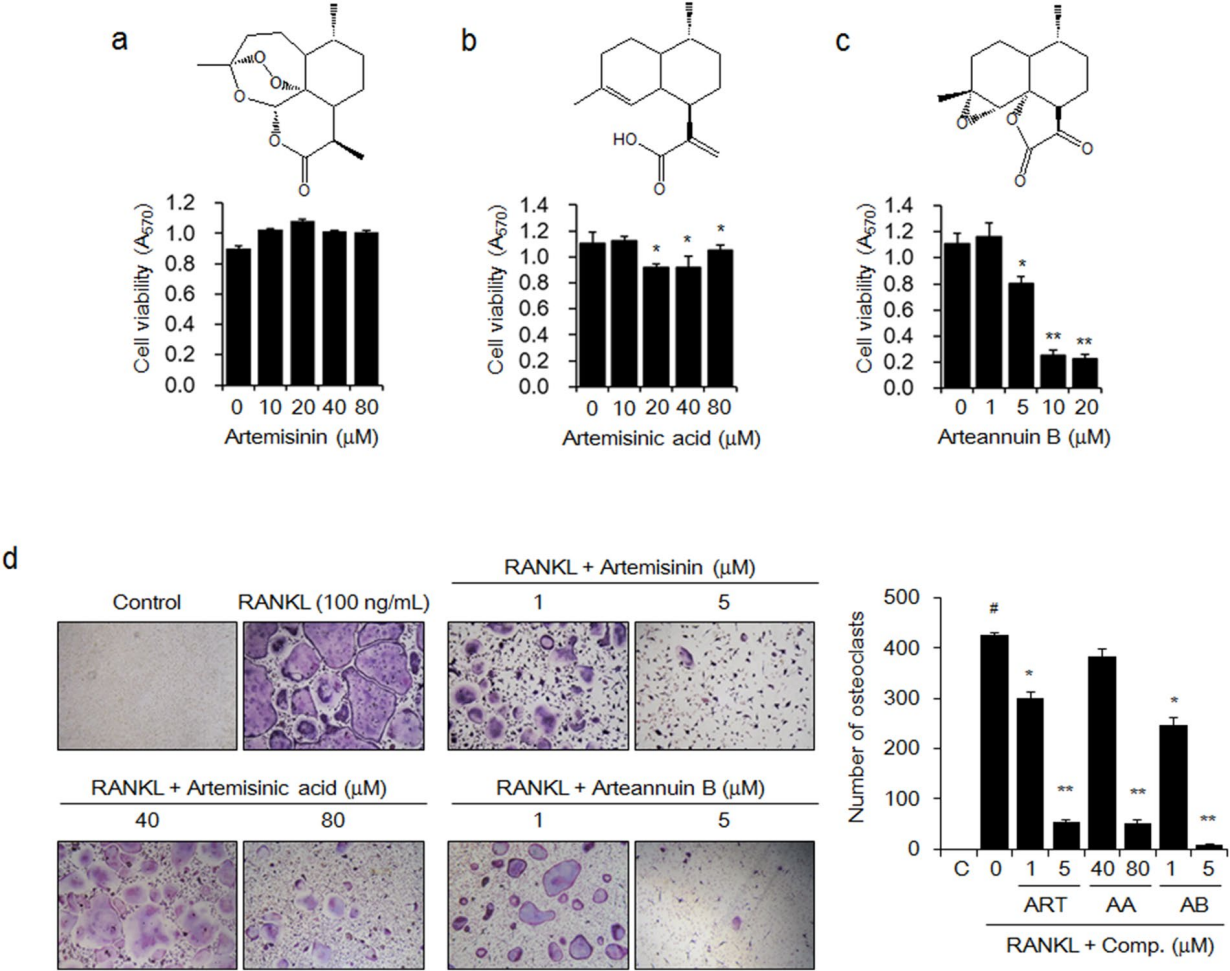
The active components of *A. annua* attenuate RANKL-induced osteoclast formation. The BMMs were cultured with M-CSF (30 ng/mL) and the indicated concentrations of (**a**) artemisinin (ART), (**b**) artemisinic acid (AA), or (**c**) arteannuin B (AB) for 5 days. Cell viability was determined using an MTT assay. The data are expressed as the mean ± SE. ^*^ *P* < 0.05, ^**^ *P* < 0.01 *versus* BMMs without active components. (**d**) BMMs were incubated in media with M-CSF (30 ng/mL), RANKL (100 ng/mL), and the indicated concentration of ART, AA, or AB for 5 days. Mature osteoclasts were detected by TRAP staining and TRAP-positive multinucleated cells were counted using light microscopy (magnification, ×100). The images are representative, and the data are expressed as the mean ± SE of triplicate experiments. ^#^ *P* < 0.01 *versus* BMMs without RANKL (C), ^*^ *P* < 0.05, ^**^ *P* < 0.01 *versus* BMMs with RANKL alone.

We assessed the anti-osteoclastic activities of artemisinin, artemisinic acid, and arteannuin B, which are known to be the active components of *A. annua* ^32^. The results of the MTT assay indicated that artemisinin (Fig. 4a) and artemisinic acid (Fig. 4b) were not cytotoxic to the BMMs at concentrations of less than 80 μM. Arteannuin B significantly decreased the viability of the BMMs at concentrations of more than 5 μM (IC_50_ = 7.3 μM) (Fig. 4c). Artemisinin and arteannuin B significantly inhibited RANKL-induced osteoclast formation by 29.5% and 41.9% at 1 μM and by 79.6% and 98.2% at 5 μM, respectively. Artemisinic acid suppressed RANKL-induced osteoclastogenesis by 87.9% at 80 μM in BMMs (Fig. 4d).

### Artemisinin, artemisinic acid, and arteannuin B inhibited mature osteoclast-mediated bone resorption

**Figure 5:**
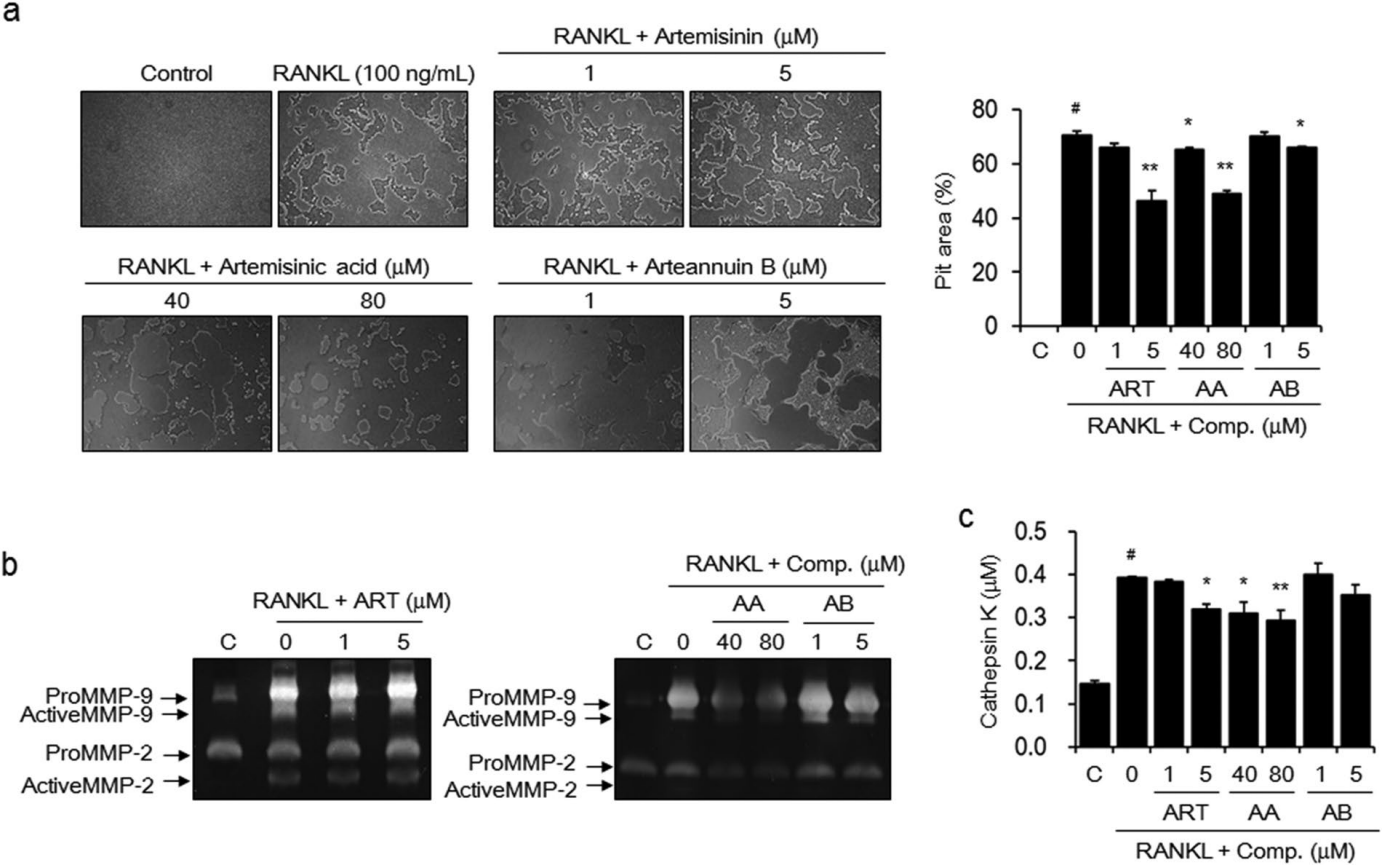
The active components inhibit the activity of RANKL-induced osteoclasts. BMMs were seeded onto Osteo Assay Surface Plates and incubated in α-MEM containing M-CSF (30 ng/mL) and RANKL (100 ng/mL) for 5 days. The cells were then exposed to the indicated concentrations of artemisinin (ART), artemisinic acid (AA), or arteannuin B (AB) for an additional 2 days. (**a**) The cells were lysed with 5% sodium hypochlorite solution, and the formed resorption pits were examined using light microscopy (magnification, ×100). The resorbed area was calculated using ImageJ software. (**b**) MMP-2 and MMP-9 activities and (**c**) cathepsin K activity were detected using gelatin zymography and a commercially available cathepsin K assay kit. The images are representative, and the data are expressed as the mean ± SE of triplicate experiments. ^#^ *P* < 0.01 *versus* BMMs without RANKL (C), ^*^ *P* < 0.05, ^**^ *P* < 0.01 *versus* BMMs with RANKL alone.

We evaluated whether artemisinin, artemisinic acid, and arteannuin B could reduce bone resorption by mature osteoclasts. BMMs were first stimulated with RANKL to induce differentiation into mature osteoclasts on an Osteo Assay Surface Plate to assess pit formation and then treated with artemisinin, artemisinic acid, or arteannuin B. Artemisinin at 5 μM and artemisinic acid at 80 μM inhibited the formation of resorption pits by RANKL-induced osteoclasts by 34.2% and 30.4%, respectively. Treatment with 5 μM arteannuin B weakly showed minimal effects on the formation of resorption pits (Fig. 5a). The activities of MMP-2 and MMP-9 in the culture media of RANKL-induced mature osteoclasts were inhibited by artemisinic acid and arteannuin B, but not by artemisinin (Fig. 5b). Cathepsin K levels were reduced by 18.3% and 25.2% in the culture media of osteoclasts treated with 5 μM artemisinin and 80 μM artemisinic acid, respectively. However, arteannuin B did not show any significant inhibitory effects (Fig. 5c).

### *A*. *annua* extract and its bioactive components suppressed the expression of key transcription factors in RANKL-induced osteoclastogenesis

**Figure 6:**
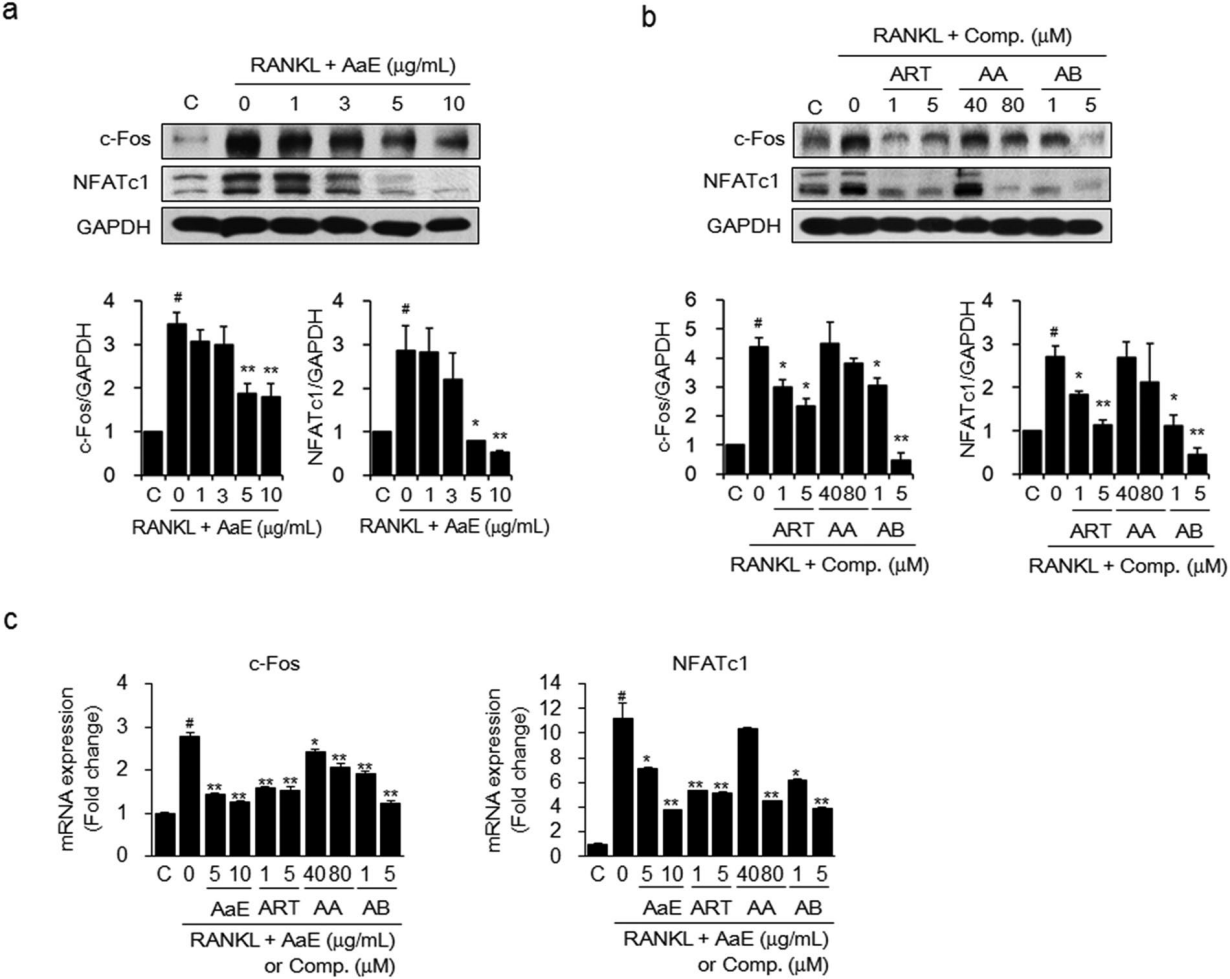
AaE and its active components suppress c-Fos and NFATc1 expression in RANKL-treated BMMs. BMMs were incubated with M-CSF (30 ng/mL), RANKL (50 ng/mL), and the indicated concentrations of (**a**) AaE and (**b**) artemisinin (ART), artemisinic acid (AA), or arteannuin B (AB) for 2 days. The expression levels of c-Fos and NFATc1 were examined by western blot analysis. The band intensity of each protein was densitometrically measured with TINA software and normalized to that of GAPDH as an internal control. The images are representative. (**c)** The mRNA expression levels of c-Fos and NFATc1 were detected using qRT-PCR and normalized to that of GAPDH. The data are expressed as the mean ± SE of triplicate experiments. ^#^ *P* < 0.01 *versus* BMMs without RANKL (C), ^*^ *P* < 0.05, ^**^ *P* < 0.01 *versus* BMMs with RANKL alone.

To determine the molecular mechanisms by which AaE and its active components inhibit the RANKL-induced differentiation of osteoclast precursor cells, we examined the expression levels of c-Fos and NFATc1, which are osteoclastogenic transcription factors^33–35^, in the cell lysates of RANKL-treated BMMs using western blot analysis. RANKL stimulation clearly increased the expression of both c-Fos and NFATc1. However, AaE treatment significantly inhibited the RANKL-induced protein expression of c-Fos and NFATc1 in a dose-dependent manner (Fig. 6a). Artemisinin and arteannuin B suppressed c-Fos and NFATc1 protein expression in the RANKL-treated BMMs but artemisinic acid did not show any significant inhibitory effects (Fig. 6b). qRT-PCR data indicated that AaE and its active components also inhibited c-Fos and NFATc1 mRNA expression (Fig. 6c).

## Discussion

In the present study, we attempted to verify the potential of AaE and its active components for the prevention and inhibition of osteoporosis. Our *in vivo* study demonstrated that oral administration of AaE and its active components, artemisinin and arteannuin B, protected against estrogen deficiency-induced bone loss by significantly blocking ovariectomy-induced alterations in bone architecture parameters, serum bone turnover markers and the pro-inflammatory cytokines TNF-α and IL-1β. According to accumulating data, estrogen deficiency-induced bone loss is widely considered to be closely linked to pro-inflammatory cytokines, including TNF-α, IL-1β, IL-6, and IL-11^36^. These pro-inflammatory cytokines have been reported to either directly or indirectly stimulate the bone-resorptive activity of osteoclasts^37^. Estrogen deficiency leads to a global increase in IL-7 production in the thymus and spleen, as well as in bone. The produced IL-7 induces TNF release from activated T cells and also stimulates T cell proliferation and activation. TNF, in turn, promotes the production of RANKL, M-CSF, IL-1, and IL-6 by stromal and osteoblastic cells, stimulating the differentiation of osteoclast precursors^31,38^. Notably, oral administration of AaE, artemisinin, and arteannuin B at the doses used in this study did not result in damage to the liver or kidney and was as effective as oral administration of 17β-estradiol. These results suggest that AaE and its major components are safe and have excellent anti-osteoporotic activity.

To support the *in vivo* anti-osteoporotic activity of AaE, we investigated the inhibitory effect of AaE on osteoclast formation and function. Two biosynthetic pathways have been reported in *A. annua*. One route leads to the production of artemisinin, and the other leads to the production of arteannuin B. Artemisinic aldehyde is biosynthesized from farnesyl diphosphate and converted to artemisinin and deoxyartemisinin via dihydroartemisinic aldehyde and dihydroartemisinic acid. On the other hand, the intermediate, artemisinic aldehyde, is oxidized to artemisinic acid, and artemisinic acid is then converted to arteannuin B^39^. Although AaE contains a number of other compounds, artemisinin, arteannuin B, and their biosynthetic precursors are considered the major active components^40,41^. In this study, we further examined anti-osteoclastic and anti-bone resorptive activities of artemisinin, arteannuin B, and artemisinic acid as the bioactive components of AaE. Treatment with AaE or these active components at non-cytotoxic concentrations reduced RANKL-induced osteoclastogenesis in the osteoclast precursors, BMMs. Osteoclast differentiation is triggered by the binding of osteoblastic/stromal RANKL to RANK in osteoclast precursors^33,42^. This binding activates the transcription factor complex AP-1 partly through induction of its critical component, c-Fos^35^. NFATc1, a key transcriptional regulator of osteoclast differentiation, is also activated by RANKL-RANK binding and subsequently enhances the expression of osteoclast-specific molecules, including TRAP and cathepsin K, in cooperation with c-Fos^34,43^. AaE, artemisinin, and arteannuin B markedly suppressed the protein and mRNA expressions of c-Fos and NFATc1, thereby inhibiting RANKL-induced osteoclastogenesis. At relatively high concentrations, artemisinic acid reduced the expression of these transcription factors. These results suggest that the anti-osteoclastic activity of AaE is due to the ability of artemisinin and arteannuin B to downregulate RANKL-induced c-Fos and NFATc1 expression.

Mature osteoclasts adhere to bone surfaces and secrete protons, which are generated by carbonic anhydrase; proteases, such as cathepsin K, that are released by lysosomes; and MMPs to stimulate the release of inorganic ions and degrade collagen in the bone^44,45^. In this study, AaE significantly inhibited the formation of resorption pits by RANKL-induced mature osteoclasts on bone-mimetic plates. AaE did not reduce the secretion of MMP-2 and MMP-9, but it decreased the production and secretion of cathepsin K. Artemisinin, arteannuin B, and artemisinic acid, also attenuated RANKL-induced pit formation. At the same concentrations, artemisinin inhibited the secretion of cathepsin K but not of MMPs. Arteannuin B did inhibit MMP-2/9 levels. Artemisinic acid decreased the activities of MMP-2/9 and cathepsin K at relatively higher concentrations. However, the anti-bone resorptive activities of these active components were not as strong as their anti-osteoclastic activities. These data reveal that the anti-bone resorptive activity of AaE may be attributed to the anti-osteoclastic activities of the three components, despite their inhibition of pit formation and bone matrix-degrading proteinase activities. In contrast, AaE and active components did not induce the activation of osteoblasts at non-cytotoxic concentrations (Supplementary Fig. S2).

Additionally, we examined the effect of AaE and the three components on the levels of osteoblast-derived RANKL and OPG. Whereas RANKL promotes osteoclast differentiation via RANK, OPG blocks osteoclast differentiation by sequestering RANKL from RANK^2^. Because estrogen deficiency-induced pro-inflammatory cytokines are directly involved in RANKL production^46^, we examined whether treatment with AaE could affect RANKL and OPG production in two human osteoblastic cell lines stimulated with either TNF-α or IL-1β (Supplementary Fig. S3). Treatment with TNF-α and IL-1β increased the secretion of both RANKL and OPG, and AaE treatment decreased the secretion of these proteins by TNF-α or IL-1β. However, the RANKL/OPG ratio was not noticeably changed by treatment with the proinflammatory cytokines and AaE. These data indicate that AaE may inhibit bone loss by inhibiting RANKL-induced osteoclast differentiation, rather than by regulating RANKL and/or OPG production in proinflammatory cytokine-stimulated osteoblastic cells.

In summary, AaE, including artemisinin, artemisinic acid, and arteannuin B, inhibited bone loss in estrogen-deficient OVX mice. AaE, artemisinin, artemisinic acid, and arteannuin B blocked RANKL-induced osteoclast differentiation via c-Fos and NFATc1 activation in precursor cells, as well as the resulting bone resorption. Taken together, our results indicate that the *in vivo* anti-osteoporotic effects of AaE may originate from the anti-osteoclastic activities of artemisinin, artemisinic acid, and arteannuin B. Therefore, AaE and its active components may be beneficial agents with high efficacy and safety for the prevention and treatment of osteoporosis and osteoclast-related bone diseases.

## Methods

### Reagents

*A. annua* extract (AaE) (MPRBE00725) was provided by the Medicinal Plant Resources Bank (http://knrrb.knrrc.or.kr, Seongnam, Korea), which is supported from the Ministry of Science, ICT, and Future Planning/National Research Foundation of Korea. Artemisinin was obtained from LKT Laboratories (St. Paul, MN), and artemisinic acid and arteannuin B were obtained from ChemFaces (Wuhan, China). Minimum essential medium-alpha (α-MEM), fetal bovine serum (FBS), phosphate-buffered saline (PBS), and antibiotics were purchased from Gibco BRL (Grand Island, NY). Recombinant mouse M-CSF and RANKL were obtained from R&D Systems (Minneapolis, MN). Dimethyl sulfoxide (DMSO), 17β-estradiol (E2), Tween-20, 3-(4,5-dimethyl-thiazol-2yl)-2,5-diphenyl tetrazolium bromide (MTT), and Histopaque-1083 were purchased from Sigma-Aldrich (St. Louis, MO). Polyclonal anti-c-Fos and anti-NFATc1 and monoclonal anti-GAPDH antibodies were obtained from Santa Cruz Biotechnology (Santa Cruz, CA). All the reagents used in this study were analytical grade.

### Animals

Four-week-old male ICR or 8-week-old female sham and ovariectomized ICR mice were purchased from NARA Biotech (Seoul, Korea) and Central Lab Animal (Seoul, Korea). The mice were provided free access to a standard chow diet (Orient, Seongnam, Korea) and tap water *ad libitum*. They were housed under specific pathogen-free conditions with a 12-h light/dark cycle and a relative humidity of 50 ± 5% at 22 ± 2 °C. All animal studies were conducted in accordance with the experimental protocols approved by Institutional Animal Care and Use Committee of the Yonsei University College of Dentistry (IACUC Approval No. 2015-0261 and 2016-0330). All methods were carried out in accordance with relevant guidelines and regulations.

### Induction of osteoporosis in OVX mice

Eight-week-old female OVX mice were divided into 4 groups of 10 mice and were administered vehicle (PBS containing 1% DMSO and 1% Tween-20), 1 or 10 mg/kg body weight (BW) of AaE, or 0.1 mg/kg BW of E2 by oral gavage 5 times per week for 12 weeks. In addition, OVX mice were divided into 6 groups of 10 mice and were administered vehicle, 10 or 20 mg/kg BW of artemisinin, 20 mg/kg BW of arteannuin B, or 0.1 mg/kg BW of E2 by oral gavage 5 times per week for 12 weeks. Sham-operated mice received the vehicle alone. Body weights were measured biweekly using an electronic scale. Blood samples were collected by cardiac puncture at the end of the experimental period, and the femora were collected. The femora were analyzed using a SkyScan 1076 μCT system (SkyScan, Aartselaar, Belgium) with an X-ray source voltage of 100 kV, a current of 100 mA, and a 0.5-mm aluminum filter as described previously^47^. The scanning angular rotation was 360°, and the angular increment was 0.5°. Two-dimensional (2D) images were generated using NRrecon software (SkyScan). After generating the 2D images, 3D microstructural images were reconstructed using Rapidform 2006 software (INUS Technology, Seoul, Korea). For the quantitative analysis of bone alteration, resident software (CTAn, SkyScan) was used to calculate the following parameters within the volume of interest: BV/TV, Tb.Th, Tb.N, Tb.Sp), and SMI.

### Determination of bone turnover marker levels in serum

The collected blood samples were allowed to clot for 2 h at room temperature and centrifuged at 2000 × g for 20 min to obtain the serum. The following kits were used to determine the levels of the bone turnover markers: the QuantiChrom Calcium Assay Kit and ALP Assay Kit (BioAssay Systems, Hayward, CA); the TRAP 5b Enzyme Immunoassay (EIA) Kit and RatLaps EIA Kit for CTX (Immunodiagnostic Systems, Fountain Hills, AZ); and the Osteocalcin EIA Kit (Biomedical Technologies, Stoughton, MA). TNF-α and IL-1β levels were quantified using their respective commercially available ELISA kits (R&D Systems). ALT, AST, BUN, and creatinine levels were quantified using EnzyChrom ALT and AST Assay Kits and the QuantiChrom Urea and Creatinine Assay Kits (BioAssay Systems).

### Histological examination

The collected femora of mice were fixed in 10% buffered formalin solution for 24 h, decalcified with 10% EDTA solution (pH 7.5) at 4 °C for two weeks and then embedded in paraffin. Serial sections (3-4 μm thick) were prepared and mounted onto slides. Hematoxylin-eosin (H&E) and TRAP staining were accomplished in the sections derived from mouse hind limbs as described previously^48^. Osteoclast surface per bone perimeter (Oc.S/BPm) values were measured with IMT i-Solution software (version 7.3, IMT i-Solution, BC, Canada) and determined as the percentage of bone surface in contact with osteoclasts.

### Cell culture

Mouse BMMs were isolated from the tibiae of four-week-old male ICR mice using Histopaque density gradient centrifugation as described previously^49^ and cultured in α-MEM containing 10% FBS and 30 ng/mL M-CSF at 37 °C in a humidified atmosphere of 5% CO_2_.

### Cell viability assay

BMMs (1 × 10^4^ cells/well) were seeded onto 96-well plates in α-MEM containing 10% FBS, 30 ng/mL M-CSF, and the indicated concentrations of AaE, artemisinin, artemisinic acid, or arteannuin B for 5 days. The cell viability was measured using an MTT assay^50^.

### Osteoclast formation assay

BMMs (5 × 10^4^ cells) were cultured in media containing 10% FBS, 30 ng/mL M-CSF, 100 ng/mL RANKL, and the indicated concentrations of AaE, artemisinin, artemisinic acid, or arteannuin B for 5 days. The cells were fixed for 1 min at room temperature, and enzyme histochemistry for TRAP was performed using an acid phosphatase leukocyte kit (Sigma-Aldrich). TRAP-positive multinuclear cells (≥3 nuclei) were counted as osteoclasts.

### Pit formation assay

Osteo Assay Surface Plates (Corning, MA) were used for the pit formation assay as described previously^50^. Briefly, BMMs (2 × 10^4^ cells) were seeded onto the plate and cultured in α-MEM containing 10% FBS, M-CSF (30 ng/mL), and RANKL (100 ng/mL) for 5 days. BMMs were then treated with either AaE, artemisinin, artemisinic acid, or arteannuin B at the indicated concentrations for an additional 2 days. The cells were lysed with 5% sodium hypochlorite solution. The images of the resorption pits were obtained using light microscopy. The resorption pit areas were measured using ImageJ software (Ver. 1.6, NIH, Bethesda, MD).

### Zymography and cathepsin K assay

The culture media and cell lysates were collected from the pit formation assay^49^ and centrifuged at 200 × g for 5 min. The activities of MMP-2 and MMP-9 were determined using gelatin zymography. The collected conditioned media were electrophoresed on 10% polyacrylamide gels containing 0.8 mg/mL gelatin. The gels were then washed with 2.5% Triton X-100 for 1 h at room temperature and incubated in a reaction buffer containing 50 mM Tris-HCl (pH 7.5), 5 mM CaCl_2_, 200 mM NaCl, and 0.02% Brij 35 at 37 °C for 24 h. After staining with 0.1% Coomassie Blue R-250, the gelatinase activity was identified as clear bands against a blue background. The cathepsin K activity in the culture media and cell lysates was determined using the SensoLyte 520 Cathepsin K Assay Kit (Anaspec, CA) according to the manufacturer’s instructions.

### Western blot analysis

BMMs (1 × 10^6^ cells) were treated with M-CSF (30 ng/mL), RANKL (100 ng/mL), and either AaE, artemisinin, artemisinic acid, or arteannuin B at the indicated concentrations for 48 h. The cells were lysed with RIPA buffer and the protein concentrations of the lysates were measured using a bicinchoninic acid (BCA) kit (Pierce, Rockford, IL). Equal amounts of protein (20 μg) were loaded onto a sodium dodecyl sulfate–polyacrylamide gel and electrophoresed. The blots were transferred to a polyvinylidene difluoride membrane (Millipore, Billerica, MA). The membrane was blocked with 5% skim milk in TBS-T (10 mM Tris, pH 8.0, 150 mM NaCl, and 0.1% Tween-20) and then incubated with primary antibodies against NFATc1 or GAPDH (1:1000) in 3% skim milk or c-Fos (1:1000) in TBS-T with 5% bovine serum albumin for 24 h at 4 °C. After washing, the blots were incubated for 1 h with secondary antibodies coupled to horseradish peroxidase (1:2000) and visualized using an ECL kit. The intensities of the bands were analyzed using TINA software (Ver. 2.0, Raytest, Straubenhardt, Germany).

### Quantitative real-time PCR

BMMs (1 × 10^6^ cells) were incubated with M-CSF (30 ng/mL), RANKL (50 ng/mL), and AaE or its active components at indicated concentrations in 60-mm dish for 48 h. Total RNA was isolated using the RNeasy Mini Kit (Qiagen, Valencia, CA) and complementary DNA was synthesized from the RNA (1 μg) by the PrimeScript RT reagent kit (Takara Bio, Otsu, Japan). Quantitative real-time PCR (qRT-PCR) was performed using the ABI 7300 Real-Time Systems (Applied Biosystems, Forster City, CA) with SYBR Premix Ex Taq^TM^ (Takara Bio) according to the manufacturer’s instructions. The qRT-PCR reaction was performed at 95 °C for 30 s, followed by 40 cycles of 95 °C for 5 s, 60 °C for 34 s. The quantity of each target was normalized to GAPDH. Primer sequences were as follows: c-Fos, forward 5′-GGCACTAGAGACG GACAGAT-3′ and reverse 5′-ACAGCCTTTCCTACTACCATTC-3′; NFATc1, forward 5′-TGTCTGTGCTCTGCTTCTC-3′ and reverse 5′-GTCTTCCGA GTTCACATCCC-3′; and GAPDH, forward 5′-GTGGAGTCATACTGGAACATGTAG-3′ and reverse 5′-AATGGT GAAGGTCGGTGTG-3′.

### Statistical analysis

Statistical analysis was performed using SPSS software ver. 20.0 (SPSS, Inc., Chicago, IL, USA). The data are expressed as the mean ± standard error (SE) and were analyzed using one-way ANOVA and Tukey test to examine the differences between the two groups. Results with *P* < 0.05 were considered statistically significant.

## Electronic supplementary material

Supplementary information
