# Supplementary material for: Artemisia annua extract prevents ovariectomy-induced bone loss by blocking receptor activator of nuclear factor kappa-B ligand-induced differentiation of osteoclasts
Source: Sci Rep. 2017 Dec 11;7:17332. doi: 10.1038/s41598-017-17427-6 (PMC5725529; doi:10.1038/s41598-017-17427-6)

Supplementary information

Artemisia annua extract prevents ovariectomy-induced bone loss by blocking receptor activator of nuclear factor kappa-B ligand-induced differentiation of osteoclasts

Sun Kyoung Lee[1], Hyungkeun Kim[1,2], Junhee Park[1,3], Hyun-Jeong Kim[1], Ki Rim Kim[4], Seung Hwa Son[5], Kwang-Kyun Park[1,2,3] and Won-Yoon Chung[1,2,3*]

[1]Department of Oral Biology, Oral Cancer Research Institute, BK21 PLUS Project, Yonsei University College of Dentistry, Seoul 03722, Korea

[2]Department of Applied Life Science, The Graduate School, Yonsei University, Seoul 03722, Korea

[3]Department of Dentistry, Graduate School, Yonsei University, Seoul 03722, Korea

[4]Department of Dental Hygiene, Kyungpook National University, Sangju 37224, Korea

[5]Department of Dental Hygiene, Gangdong College, Icheon, 27600, Republic of Korea

Supplementary methods

Cell viability assay. MC3T3-E1 osteoblastic cells (5×10^3 cells/well) were seeded onto 6-well plates with 10% FBS-α-MEM for 24 h. The cells were incubated in serum-free α-MEM with AaE, artemsinin, artemisinic acid, or arteannuin B at the indicated concentrations for 24 h and 72 h. The cell viability was measured using an MTT assay.

Alkaline phosphatase activity. MC3T3-E1 cells (5×10^4 cells/well) were seeded onto 12-well plates with 10% FBS-α-MEM (growth medium) or 10% FBS-α-MEM containing 10 nM dexamethasone, 50 μg/mL ascorbic acid, and 2 mM β-glycerophosphate (osteogenic medium). The cells were treated with the indicated concentrations of AaE, artemisinin, artemisinic acid, or arteannuin B for 72 h. To assess the activity of alkaline phosphatase, the cells were washed with PBS and then suspended in 0.5 mL PBS containing 0.1 M glycine, 1 mM $MgCl_2$, and 0.05% Triton X-100. The lysate (100 μL was incubated with 250 μL of *p*-nitrophenyl phosphate solution at 37°C for 30 min. Enzymatic activity was terminated by adding 100 μL of ice cold 3 M NaOH, and the amount of liberated *p*-nitrophenol was measured by monitoring absorbance at 405 nm.

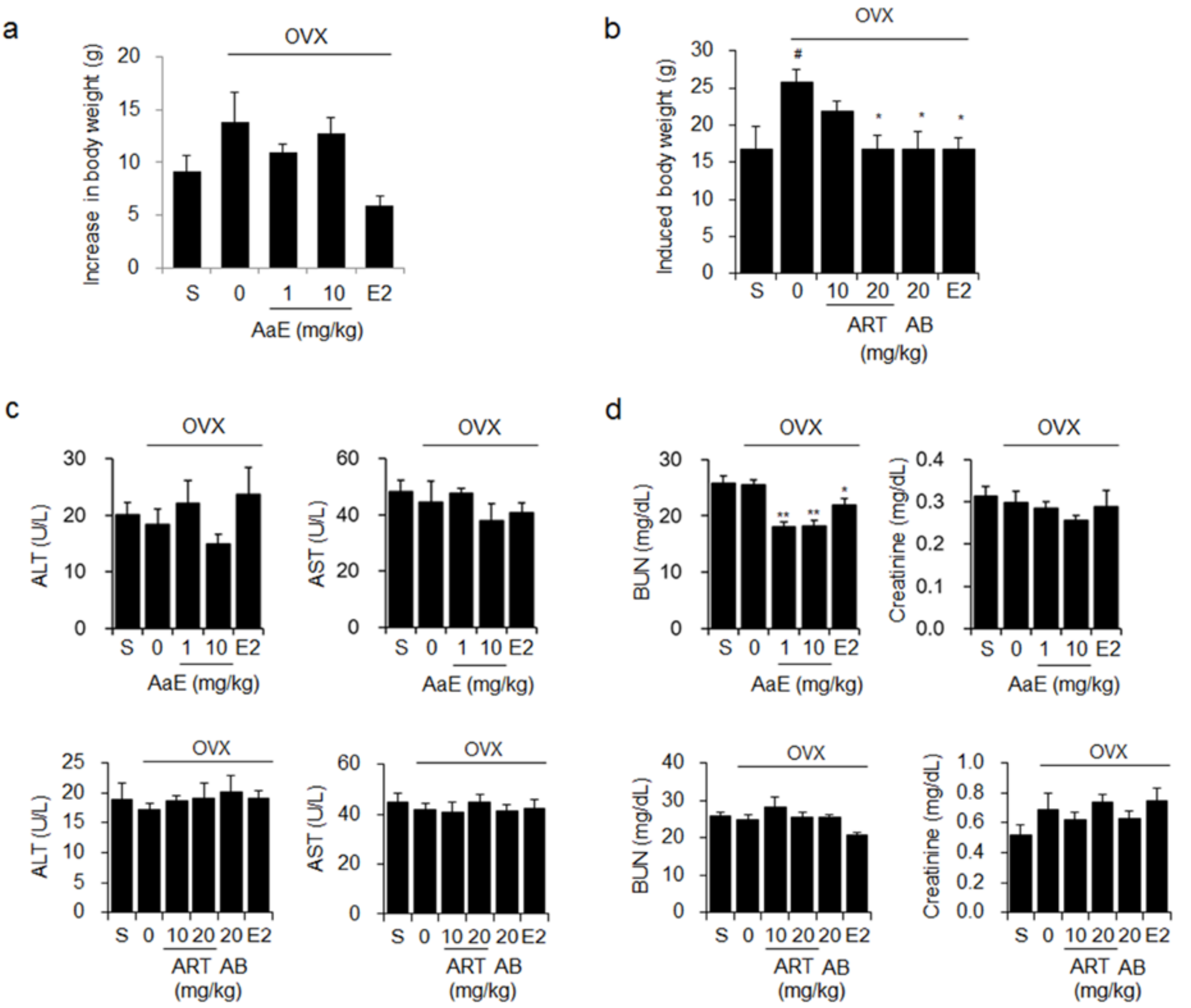

Figure S1. The effects of AaE, artemisinin, and arteannuin B on body weight and normal tissues in OVX mice. Eight-week-old female OVX mice (N=10) were administered vehicle (PBS containing 1% DMSO and 1% Tween-20), 1 or 10 mg/kg BW of AaE, or 0.1 mg/kg BW of E2 by oral gavage 5 times per week for 12 weeks. In addition, OVX mice were divided into 6 groups of 10 mice and were administered vehicle, 10 or 20 mg/kg BW of artemisinin, 20 mg/kg BW of arteannuin B, or 0.1 mg/kg BW of E2 by oral gavage 5 times per week for 12 weeks. Sham-operated mice (N=10) received the vehicle alone. The body weights were measured in mice treated (a) AaE, (b) artemisinin (ART) or arteannuin B (AB) at 12 weeks. Serum levels of (c) ALT and AST to assess liver damage and (d) BUN and creatinine to assess kidney damage were quantified using their respective commercially available assay kits. The data are expressed as the mean ± SE. $^{\#}P < 0.01$ *versus* sham-operated mice (S), $^{*}P < 0.05$, $^{**}P < 0.01$ *versus* OVX mice.

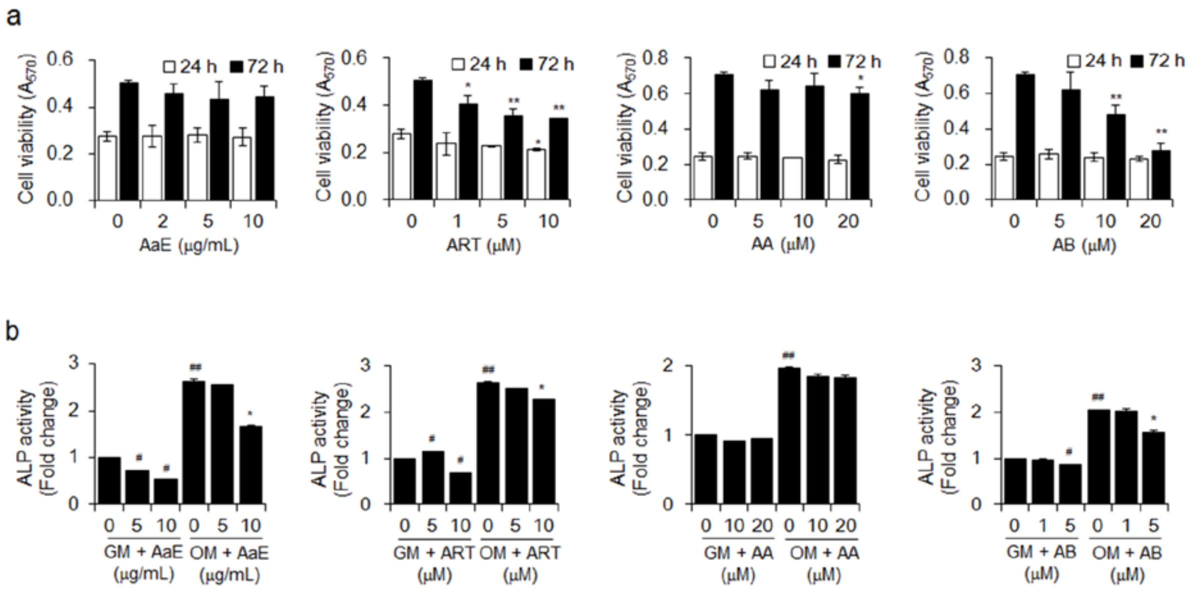

Figure S2. The effects of AaE and its active components on ALP activity of osteoblastic cells. (a) MC3T3-E1 cells were incubated with the indicated concentration of AaE, artemisinin (ART), artemisinic acid (AA), or arteannuin B (AB) for 24 h and 72 h. Cell viability was analyzed using an MTT assay. The data are expressed as the mean ± SE. $^{*}P < 0.05$, $^{**}P < 0.01$ *versus* cells without compounds. (b) MC3T3-E1 cells were cultured in growth medium (GM) or osteogenic medium (OM) treated with the indicated concentration of AaE, ART, AA, or AB for 72 h. ALP activity was assessed as described in the *Supplementary methods* section. The data are expressed as the mean ± SE. $^{\#}P <$ 0.05, $^{\#\#}P < 0.01$ *versus* cells with GM alone, $^{*}P < 0.01$ *versus* cells with OM alone.

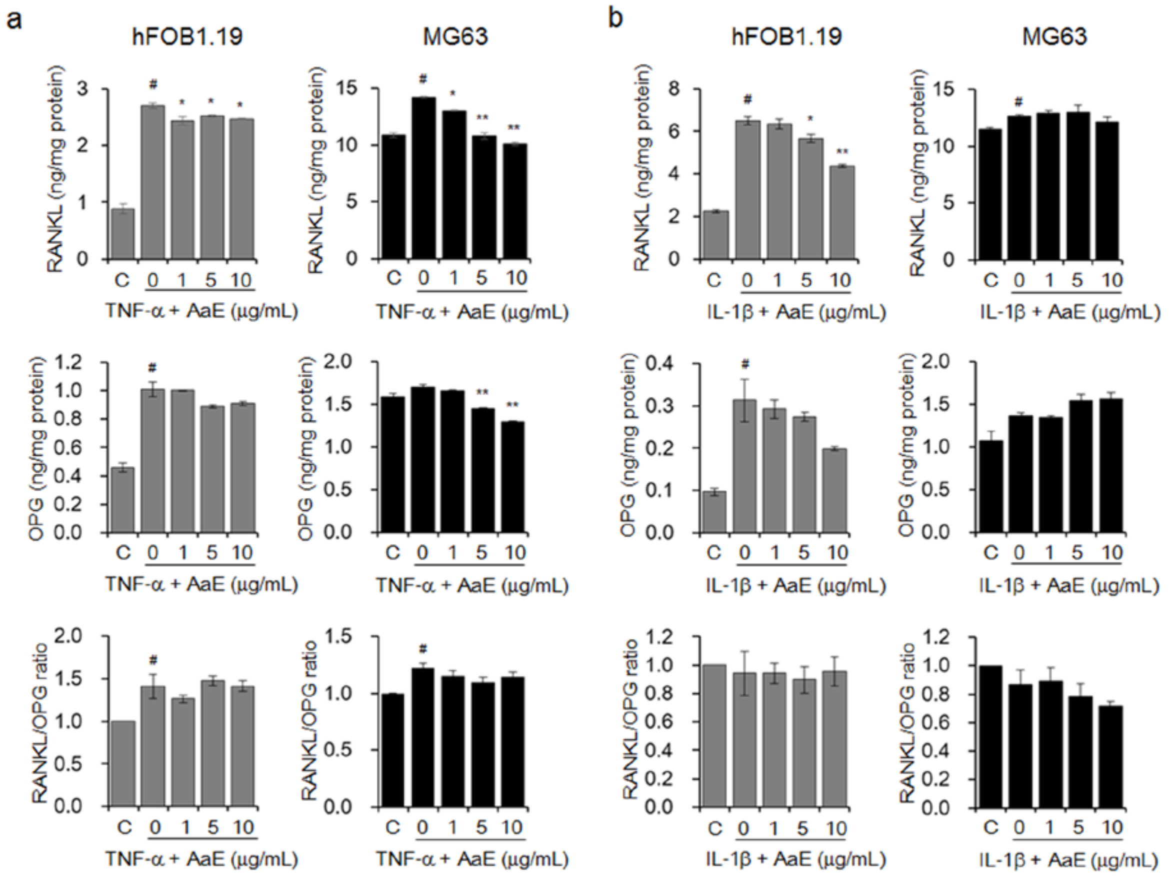

Figure S3. The effects of AaE on RANKL and OPG secretion from proinflammatory cytokine-stimulated osteoblastic cells. hFOB 1.19 and MG-63 cells were incubated with (a) TNF-α (10 ng/mL) or (b) IL-1β (10 ng/mL) and the indicated concentration of AaE for 24 h. RANKL and OPG levels in culture media from the osteoblastic cells were measured using commercially available RANKL and OPG ELISA kits. The data are expressed as the mean ± SE. $^{\#}P < 0.05$ *versus* cells treated with media alone (C), $^{*}P<0.05$, $^{**}P<0.01$ *versus* TNF-α-treated cells or IL-1β-treated cells.

Figure 3d

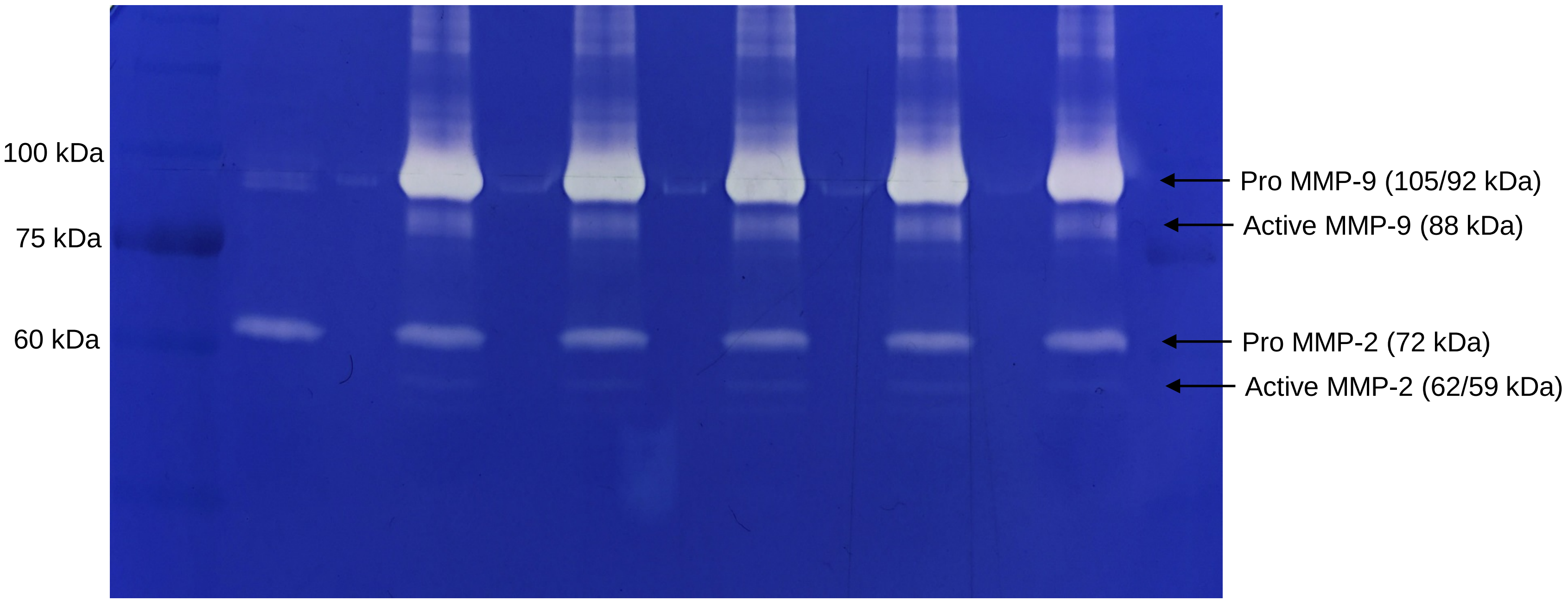

100 kDa
75 kDa
60 kDa
Pro MMP-9 (105/92 kDa)
Active MMP-9 (88 kDa)
Pro MMP-2 (72 kDa)
Active MMP-2 (62/59 kDa)

Figure 5b

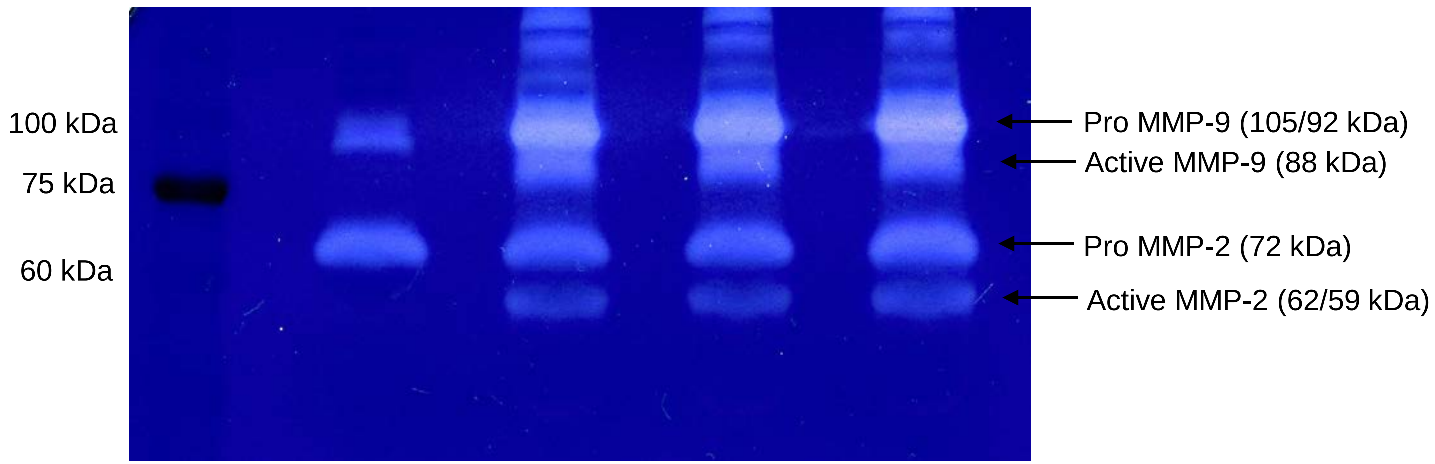
100 kDa
75 kDa
60 kDa
Pro MMP-9 (105/92 kDa)
Active MMP-9 (88 kDa)
Pro MMP-2 (72 kDa)
Active MMP-2 (62/59 kDa)

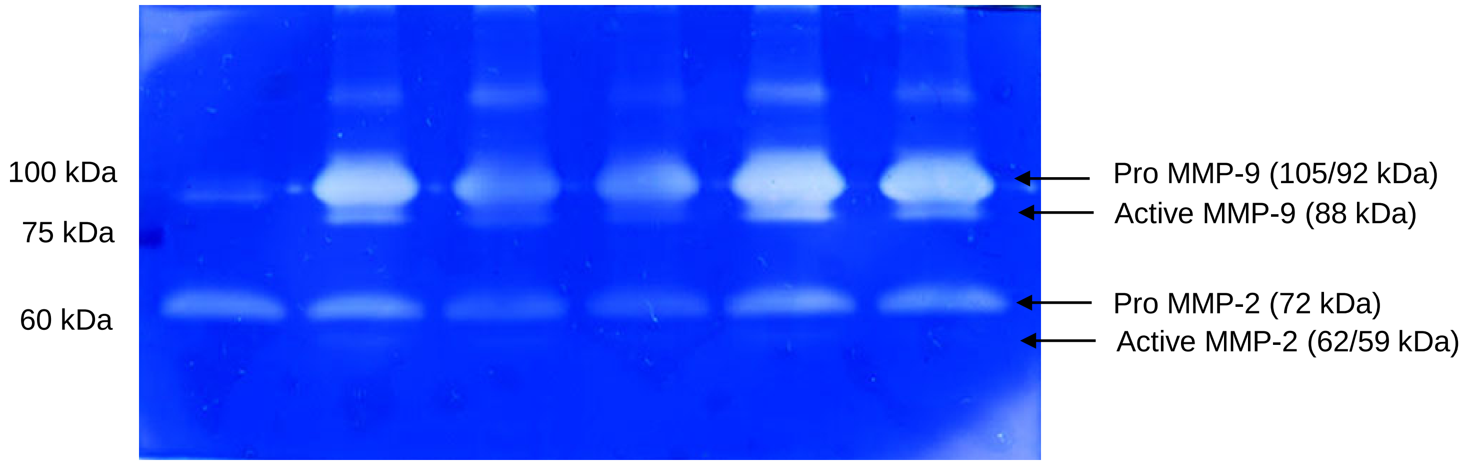
100 kDa
75 kDa
60 kDa
Pro MMP-9 (105/92 kDa)
Active MMP-9 (88 kDa)
Pro MMP-2 (72 kDa)
Active MMP-2 (62/59 kDa)

Figure 6a

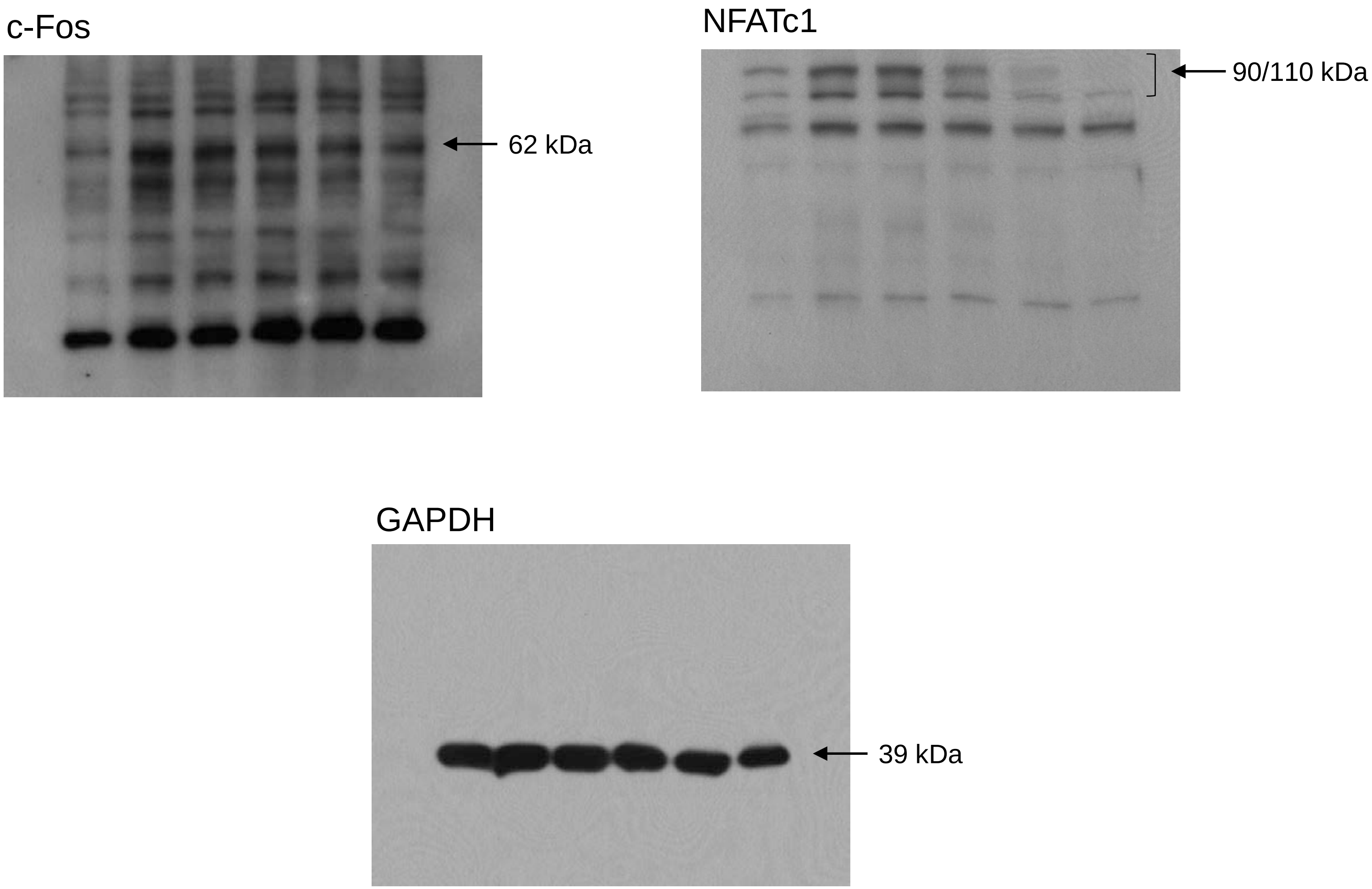

Figure 6b

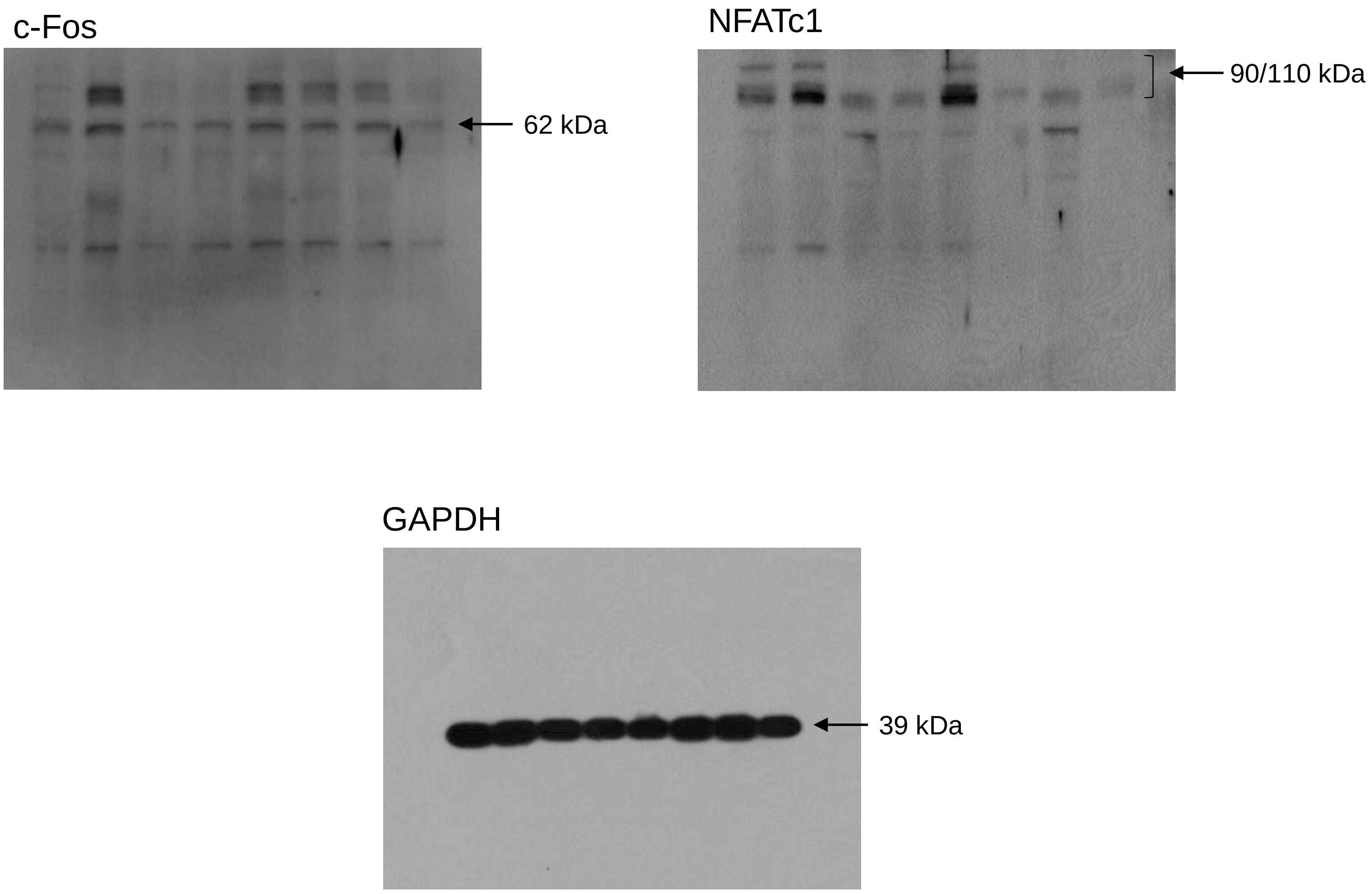

Supplement: Supplementary file 1 — Supplementary information [file 41598_2017_17427_MOESM1_ESM.pdf]
